# Accessing the Digital Health Application (DiGA) Market: Key Success Factors, Market Barriers and Strategies for Sustainable Adoption

**DOI:** 10.1177/00469580261433432

**Published:** 2026-04-10

**Authors:** Lukas Schramm, Nadine Cebulla, Christian Greis, Claus-Christian Carbon

**Affiliations:** 1 University of Bamberg, Bavaria, Germany; 2 University Hospital Würzburg, Germany; 3 University Hospital Zurich, Switzerland

**Keywords:** digital health, DiGA, digital supply act, digital health applications, digital therapeutics, mHealth, ehealth, entrepreneur, leadership, DTx, success, digitalization

## Abstract

Although the digital health application (DiGA) market in Germany offers great opportunities for developing powerful digital applications, it has also faced substantial challenges. DiGA are evidence-based digital interventions in Germany that have been established as reimbursable solutions. This initiative is being copied in other European countries, which have introduced similar pathways. This study systematically identifies and assesses key success factors, marketing and sales strategies and critical DiGA components affecting DiGA adoption. A mixed-methods approach was applied, combining semi-structured expert interviews (*n* = 32) with a quantitative survey (*n* = 142) among DiGA manufacturers, healthcare professionals, and industry experts. Statistical comparisons between high prescription volumes (>15 000), moderate prescription volumes (2000-15 000) and low prescription volumes (<2000) DiGA manufacturers were conducted to determine significant differences in factor scoring. The findings highlight three core drivers of DiGA success: robust clinical evidence, access to the right physicians, and awareness at the point of care. High regulatory burdens, the DiGA prescription process, and physicians’ unawareness were identified as the greatest barriers to adoption. The indication-specific nature of DiGA success was evident, emphasizing the need for tailored go-to-market strategies. This study provides the first structured and data-driven analysis of DiGA commercialization success factors and major challenges. The results offer strategic guidance for manufacturers, emphasizing strategic prescriber access and regulatory adaptation as key enablers of sustainable adoption. Future research should further explore indication-specific strategies, regulatory impact assessments, and international validation to enhance DiGA scalability and integration into healthcare systems.

## Introduction

### The Present Research Approach

The digital transformation of healthcare is reshaping the delivery and access of medical services.^1^ Mobile health (mHealth) technologies and digital therapeutics (DTx) have emerged as pivotal tools, enabling personalized care and enhancing patient engagement.^2^ A DiGA (Digitale Gesundheitsanwendung, i.e., digital health application) is a special category of digital health tool officially recognized and regulated in Germany, typically implemented as a smartphone app or browser-based web application.^3,4^ The DiGA ecosystem has been proven to offer powerful and efficient patient support in the detection, monitoring, treatment, or alleviation of illnesses, injuries, or disabilities through, for example, therapeutic elements, education, and behavioral changes.^3-6^ DiGA can be prescribed by physicians or psychotherapists or requested directly by insured patients with the corresponding condition.^3,4,6-8^ They integrate seamlessly into the German healthcare systems, offering evidence-based interventions and treatment.^8^

The international interest in DiGA is increasingly gaining momentum, as evidenced by the German Federal Institute for Drugs and Medical Devices (Bundesinstitut für Arzneimittel und Medizinprodukte, BfArM), sharing its experiences with countries such as Denmark, Belgium, the Netherlands, Austria, France, Finland, the United Kingdom, the United States, and Korea, indicate a vibrant exchange on both institutional and project-specific levels.^9-13^ Particularly, foreign authorities show keen interest in Germany’s expedited approval process (“Fast-Track”) for DiGA.^13^ Unfortunately, the specific country requirements for digital health applications and digital medical devices still lead to increased costs and resources. Therefore, new plans to harmonize market access and reimbursement seem to be promising.^9,10,13,14^

With the introduction of the Digital Healthcare Act in Germany in 2019, the costs of prescribing DiGA are covered by statutory health insurance.^5,8^ The first DiGAs have been on the market since September 2020, but the number of new DiGAs listed by the BfArM is decreasing for 2024.^15,16^ One of the reasons given for this trend might be the constantly growing requirements on DiGAs.^7,17,18^ Furthermore, DiGA manufacturers who have listed their application in the DiGA directory (provisionally) successfully are still threatened by removal from the directory due to a lack of proof of benefits for permanent listing.^16^ Also, low prescription figures and high costs are leading to insolvency.^19-21^ The insolvency announcement by DiGA manufacturer Perfood with 2 provisionally listed DiGAs is just 1 example of this.^20^ Current trends assume a consolidation of the market and market access by medium-sized and large pharmaceutical and medtech companies.^22,23^ Two examples are the acquisition of the DiGA’s Zanadio^®^ and Pink!^®^ by Sidekick or the acquisition of the insolvent CaraCare^®^ by Bayer.^22,23^ Based on the last report of the National Association of Statutory Health Insurance Funds (GKV), 10 out of 68 most prescribed DiGAs account for 71% of total prescriptions.^3^ In the most recent report, dated April 1, 2025, it was found that the 3 DiGA with the most prescriptions accounted for 38% of the total.^3^ This figure is derived from the assessment period between the DiGA introduction in September 2020 and December 31, 2024.^3^ So, how does the big difference in prescription figures arise, and what is needed to successfully market DiGA? Can other countries perhaps learn from Germany being a pioneer in this respect?

## Research Design and Methods

To identify critical success factors and major challenges in the distribution of DiGA, semi-structured expert interviews were conducted. Based on these qualitative semi-structured interviews, an online survey was developed to weight and rank the subjective assessments provided by the experts.^24^ This quantitizing approach was aimed to verify and objectify the qualitative findings through quantitative data.^25^

### Data Collection via Semi-structured Expert Interviews

The interview guideline was developed collaboratively by methodological and digital health experts and applied in accordance with established methodological standards for semi-structured expert interviews in qualitative health research (see Supplemental S2). Semi-structured interviews were employed to balance standardization and flexibility. Key questions were posed uniformly to all participants, ensuring comparability, while individualized follow-up questions allowed for deeper exploration.^26^ The questionnaire development and validation process is described in detail in Supplemental S2.

Interview participants were identified by analyzing publicly available CVs and accessible profiles of DiGA founders. The selection process prioritized professionals with significant experience in the distribution of DiGA, ensuring relevance to the study’s objectives and focusing on the most successful DiGA manufacturers based on the number of prescriptions. Additional industry professionals like top consultants, scientists in the field of digital health and companies that made DiGA distribution their profession. To ensure comparability across interviews, interview outcomes were standardized during data analysis through a systematic inductive coding and thematic consolidation process. Semantically similar statements were merged, duplicates were removed, and recurring themes were iteratively consolidated across interviews prior to survey development.

A sample size of *n* = 32, within the relatively limited context of DiGA manufacturers, offered a broader and more representative basis than what is found in existing research on this topic. Details of the sample are provided in the Results section. Microsoft Teams was used for conducting interviews and transcription. See Supplementals S1 and S2 for details.

### Creation of the Survey

A survey was developed based on the results of the semi-structured interviews to gather quantitative data. We analyzed the interview results after 32 interviews to identify the most frequently mentioned responses for each question. Results were obtained through a thematic qualitative analysis of transcribed expert interviews, using an inductive coding approach to identify recurring patterns and statements. Responses that were highly indication-specific or mentioned only once or twice were excluded. Duplicates and semantically similar responses were then consolidated through iterative categorization as described earlier. These consolidated answers were then used to formulate the response options for the survey, ensuring alignment with the qualitative findings.

A pretest phase aimed to validate the questionnaire’s completeness, comprehensibility and processing time, ensuring its effectiveness in gathering accurate and meaningful data.^27^

LimeSurvey^®^ was selected for this study due to its compatibility with the above-mentioned characteristics. The data from LimeSurvey^®^ was analyzed using SPSS^®^ version 29.0 and visualized using Microsoft Excel and GraphPad Prism^®^ version 10.04.01 to determine significance. A metric distribution was assumed for the evaluation in order to carry out pot-hoc tests.

### Data Collection via Survey

The recruitment of survey participants employed an active strategy by screening further online profiles and leveraging the contacts of interview partners in a snowball sampling methodology. In particular, the interviewees themselves take part in the survey again, a deliberate measure, on the one hand, to share the results with all experts and, on the other hand, to be able to compare supplemented answer options with their own. The disadvantage of this approach was possible systematic errors, including under- and over-coverage. The target population primarily encompasses personnel affiliated with DiGA manufacturers, consultants advising these entities and DiGA-affine prescribers.

The survey was slated for execution in the last quarter of 2024. The Interviews were conducted in the initial quarter of 2024.

## Results

We screened a total of 229 profiles and excluded those without professional connection to the development or commercialization of DiGA, had insufficient seniority, redundancies, or unverifiable information. Among the 54 experts we invited, several declined due to time constraints, lack of interest, or remuneration demands that were incompatible with the framework of the study. Ultimately, 32 experts participated in the interviews. The demographics of the participants are displayed in Table 1.

**Table 1.** Demographics of Interview Partners.

Participant	Sex	Age	Job title	Digital health experience	DiGA experience	DiGA manufacturer	Duration, mm:ss
I1	m	55	Consultant	27	3	n	37:37
I2	m	49	CEO	16	3	n	36:56
I3	m	31	Consultant	5	4	n	27:25
I4	m	32	COO	2	2	y	43:16
I5	m	33	Business developer	5	3	y	34:53
I6	m	56	Consultant	5	3	n	52:14
I7	m	46	CEO	12	3	y	17:59
I8	m	32	Head of market access	4	4	y	37:07
I9	w	43	Consultant	13	3	n	36:35
I10	m	41	CEO	5	4	y	31:38
I11	m	33	Director	9	2	n	28:33
I12	m	30	Head of strategic projects	4	4	y	27:52
I13	w	41	Senior manager	9	9	y	45:10
I14	w	35	Manager	10	2	n	18:51
I15	m	40	Business developer	4	4	n	25:21
I16	m	46	Head of digital Health	3	3	y	19:38
I17	m	38	CEO	20	5	n	26:19
I18	m	60	CDO	10	5	n	22:34
I19	m	54	Consultant	16	3	n	42:17
I20	m	37	Consultant	4	3	n	21:34
I21	m	43	Consultant	10	5	n	33:48
I22	w	31	Consultant	5	5	n	32:33
I23	m	60	Director	10	5	n	29:38
I24	m	25	Founder	2	2	n	28:48
I25	w	26	Consultant	4	3	n	10:01
I26	m	37	Consultant	10	4	n	32:03
I27	w	61	Physician	20	5	n	24:44
I28	w	31	Senior manager	8	4	n	16:09
I29	m	60	CEO	35	5	n	44:25
I30	m	40	CEO	4	4	n	27:36
I31	w	31	Product owner	5	5	y	27:46
I32	m	46	Manager	2	2	y	56:01

Of the 32 interview participants, 24 (75%) were male and 8 (25%) female. The average age was 41.3 years. Participants included 10 DiGA manufacturers, 10 professional consultants specializing in DiGA development, regulation, or commercialization, and 12 physicians and researchers with relevant expertise in digital health. The median experience in the digital health sector was 6.5 years (min = 2 years, max = 35 years), and the median experience with DiGA was 4 years (min = 2 years, max = 9 years). The interviewee explained the deviation in DiGA experience, with 9 years of DiGA experience, although DiGA has only been around for 5 years, with her involvement in DiGA legislation. The average duration of the interview was 31 min and 10 s.

### Interview Outcomes

The interviews identified a total of 193 success factors for DiGA distribution (1). One hundred and twenty-three marketing and sales strategies for effective market positioning (2) and 153 key components or features relevant to DiGAs and their manufacturers (3) were identified. Furthermore, 155 barriers to DiGA distribution (4) were reported, along with 135 future scenarios (5) derived from these barriers and broader market observations.

### Selection and Consolidation of Survey Items

**Figure 1.**
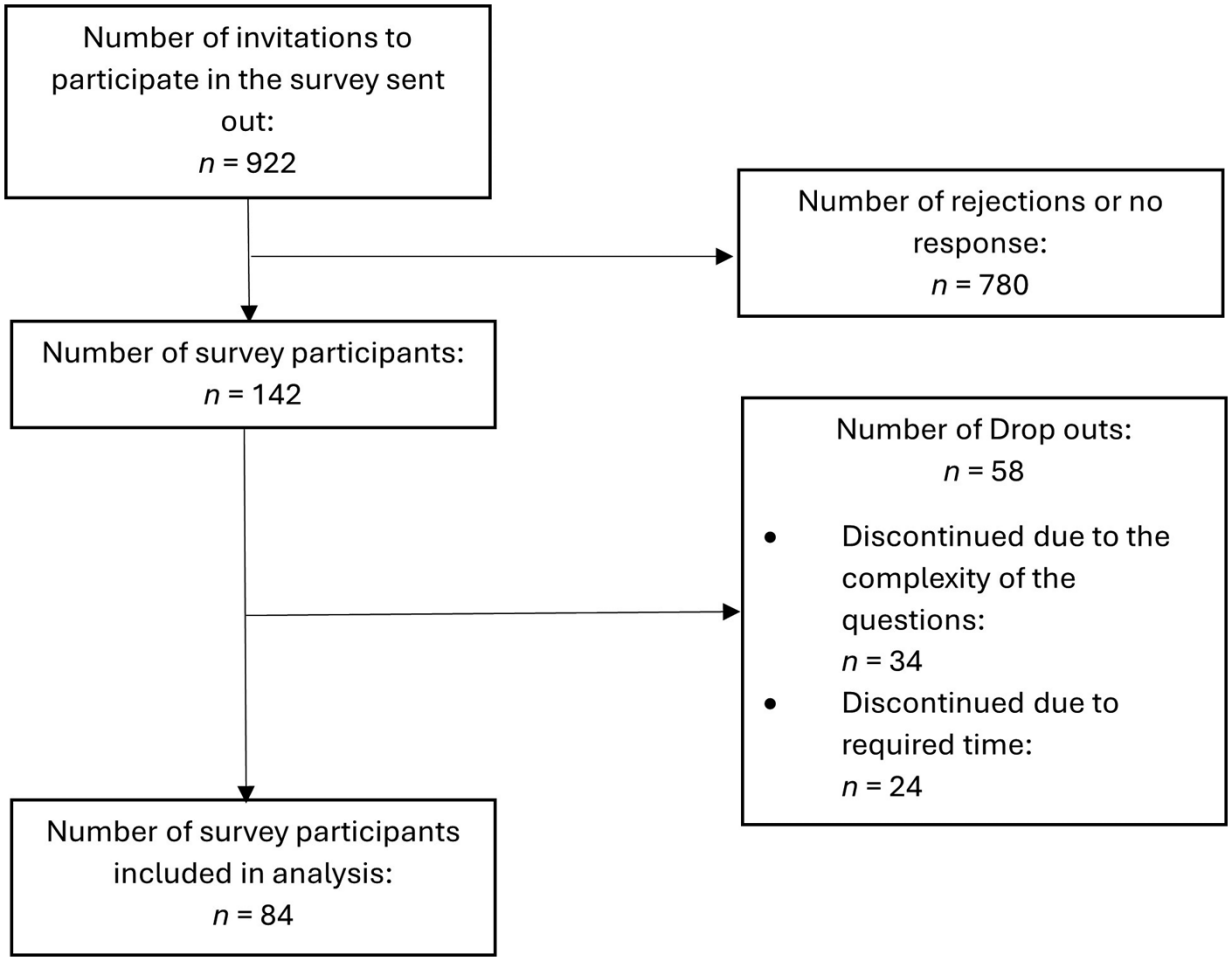
Flowchart showing survey participation and response characteristics.

Following the initial consolidation, semantically similar statements were grouped and unified into consolidated factors. The frequency of these consolidated factors was then assessed across interviews, and only those mentioned by at least three experts were retained. Using this approach, the initial set of success factors was first reduced to 65 and subsequently to a final set of 22 items evaluated in the questionnaire. The same consolidation and frequency-based selection procedure was applied analogously to marketing and sales strategies, key components, challenges, and future scenarios. For marketing and sales strategies, the responses were narrowed down to 45, of which 9 were included in the survey. Key components were consolidated to 54, with 12 selected for inclusion. Similarly, challenges were reduced to 53, with 11 included in the final survey. Future scenarios were refined to 39, with 15 scenarios incorporated into the questionnaire. This structured selection ensured a focus on the most frequently cited and relevant responses. Figure 1 demonstrates the selection process by means of a flowchart.

### Survey Participation and Response Characteristics

**Figure 2.**
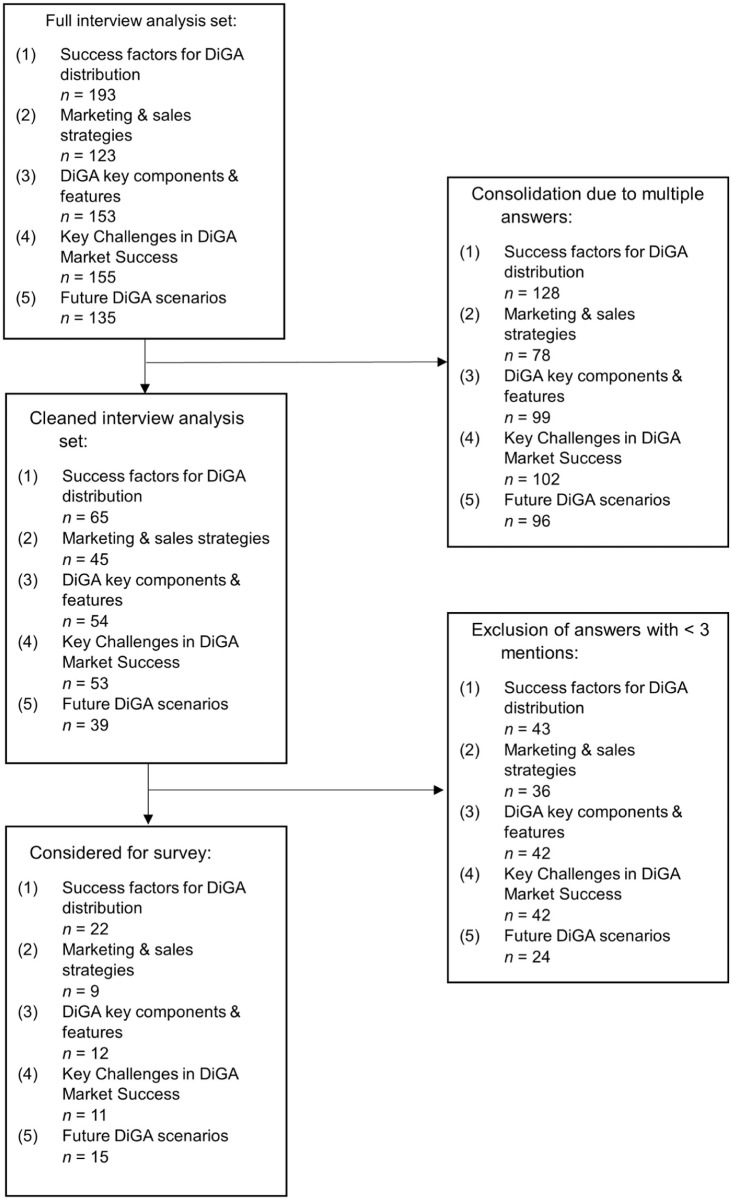
Flowchart showing the selection of answers given in the survey.

A total of *n* = 922 individuals were actively invited to participate in the survey through digital health events, business media, and professional networks. Given the niche nature of the DiGA sector, *n* = 142 participants responded to the survey. However, due to the complexity of the questions and the required level of expertise in legal, technical, and DiGA industry-specific considerations, *n* = 34 participants discontinued the survey at an early stage. Another *n* = 24 dropped out later, mainly due to the survey’s length. Ultimately, *n* = 84 participants fully completed the survey. In Table 2 below is a detailed breakdown of the full analysis set of participant characteristics. Figure 2 shows a flowchart illustrating the answers given in the survey.

**Table 2.** Survey Participant Characteristics (FAS).

Gender	Male	Female	Divers	n/a		
	*n* = 51	*n* = 32	*n* = 0	*n* = 1		
Age group	18-30 years	31-40 years	41-50 years	51-50 years	>60 years	
	*n* = 11	*n* = 35	*n* = 22	*n* = 14	*n* = 2	
Profession	Consultant	Physician	DiGA-Manufacturer	Other		
	*n* = 34	*n* = 11	*n* = 20	*n* = 19		
Experience in the digital health	<1	1-3 years	3-5 years	5-10 years	More than 10 years	No experience
	*n* = 4	*n* = 23	*n* = 21	*n* = 18	*n* = 16	*n* = 2
DiGA experience	<1	1-3 years	3-5 years	No DiGA experience		
	*n* = 9	*n* = 36	*n* = 35	*n* = 4		
DiGA manufacturer’s operating result	Positive	Negative	Secret			
	*n* = 2	*n* = 13	*n* = 5			
DiGA manufacturer 2023 prescriptions	>20 000	15 000-20 000	10 000-15 000	2000-10 000	<2000	
	*n* = 4	*n* = 2	*n* = 5	*n* = 1	*n* = 8	

#### Key success factors of DiGA distribution

The survey results indicated that clinical evidence, existing professional networks, and awareness at the point of care were identified as the most critical factors for successful DiGA distribution. Each of these factors received a median rating of 9 out of 10, with 1 indicating irrelevance and 10 indicating *essential importance*.

**Figure 3.**
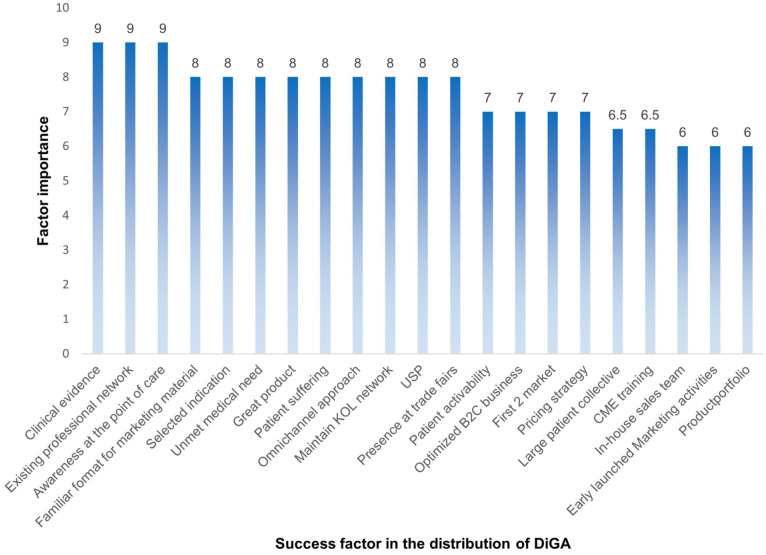
Success factors for DiGA distribution – overview.

1. Clinical evidence.
  Repeated confirmation of clinical efficacy with recognized endpoints was deemed fundamental for credibility and acceptance within the medical community. Interviewees emphasized that robust clinical validation enhances trust among healthcare professionals, regulators, and payers, facilitating prescription and reimbursement processes.
2. Existing professional networks
  The strength of pre-existing strong networks within the healthcare sector was highlighted as a key driver of success. Establishing trusted relationships with specialists and key stakeholders not only fosters long-term collaboration but also facilitates access to new markets.
3. Awareness at the point of care
  The survey results underscored the importance of strong presence, targeted educational initiatives, scientific publications, conference presentations, journal articles, explainer videos, and even traditional channels such as fax-based communication to increase prescriber awareness.
  Beyond these three top-rated factors, several additional elements were rated highly, receiving a median score of 8 out of 10. Further information on this and other factors, including scoring and definition, can be found in the Supplemental S3 and Figure 3:

#### Key Marketing & Sales Strategies for DiGA Distribution

The survey results identified access to physicians as the most critical marketing strategy, receiving a median rating of 9 out of 10. The strategy was defined as follows:

1. Access to Prescribers 
  Choosing the proper access to prescribers and providing evidence-based information through publications, conferences, and practice visits enhances trust and visibility.

In addition to this top-ranked marketing and sales strategy, 3 further approaches were rated highly, each receiving a median score of 8 out of 10:

2. Prescriber targeting 
  A marketing approach focused on the appropriate specialty group ensures that DiGA solutions reach the most relevant prescribers.
3. Improvement in conversion rates 
  Enhancing the efficiency of the prescription process through prescription services, telemedicine integrations, diagnostic assistance tools, or appointment booking systems.
4. Optimized patient journey
   Aligning marketing efforts with the patient journey to ensure engagement at the most relevant touchpoints.

**Figure 4.**
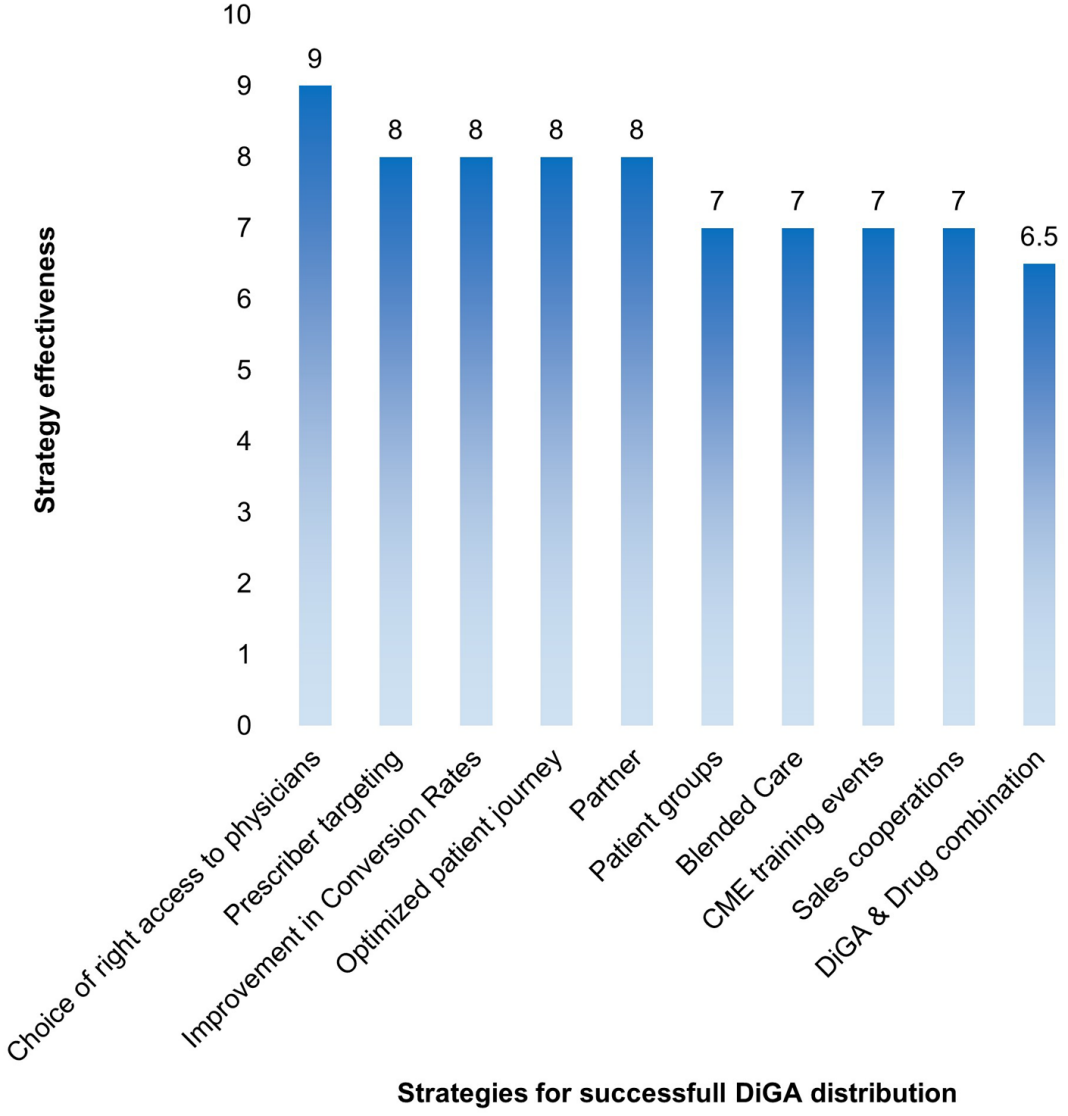
Marketing and sales strategies for DiGA distribution – overview.

Further marketing and sales strategies can be explored in Supplemental S3 and Figure 4:

#### Key Components and Features of Successful DiGA Manufacturers

The survey results identified patient success and a great product as the most critical feature and product characteristic for DiGA manufacturers, receiving a median rating of 9 out of 10. These factors were defined as follows:

1. Patient success 
  Demonstrating patient success, value, and effectiveness to prescribers and patients encourages acceptance and long-term use of the DiGA.
2. Great product 
  A user-centered design with stable functionality, intuitive usability, motivational elements that enhance adherence, and personalized features supported by gamification and a well-structured user journey.

**Figure 5.**
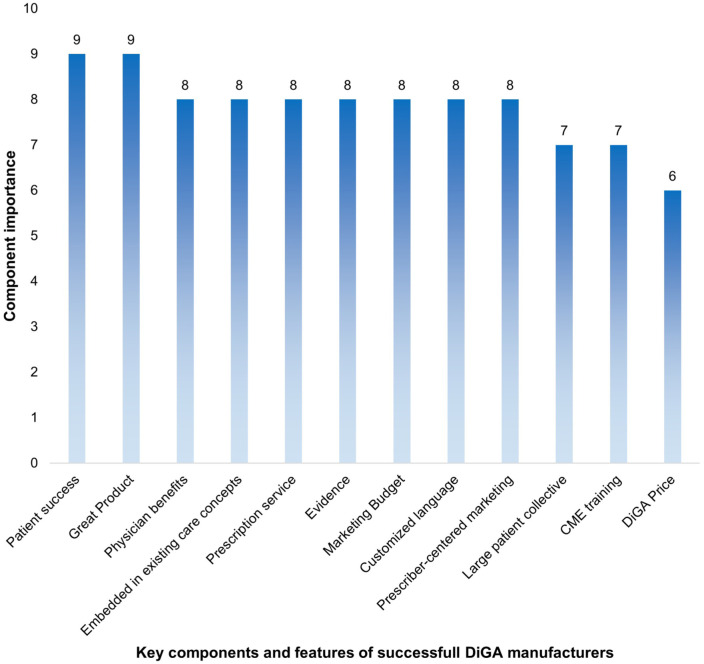
Key components and features of successful DiGA manufacturers – overview.

An overview of further key components and features of success DiGA is defined in the Supplemental S3 and Figure 5:

#### Key challenges in DiGA market success

The survey results identified growing regulatory requirements as the most critical challenge for DiGA manufacturers, receiving a median rating of 9 out of 10. This challenge was defined as follows:

1. Regulations 
  Growing regulatory requirements for studies, data protection, information security and interoperability.

Closely following in second place, with a median rating of 8.5, is:

2. Prescribers’ ignorance 
  Many prescribers are unaware of or resistant to DiGA solutions, leading to slow market penetration with only early adopters reached, while the majority remains untapped.

**Figure 6.**
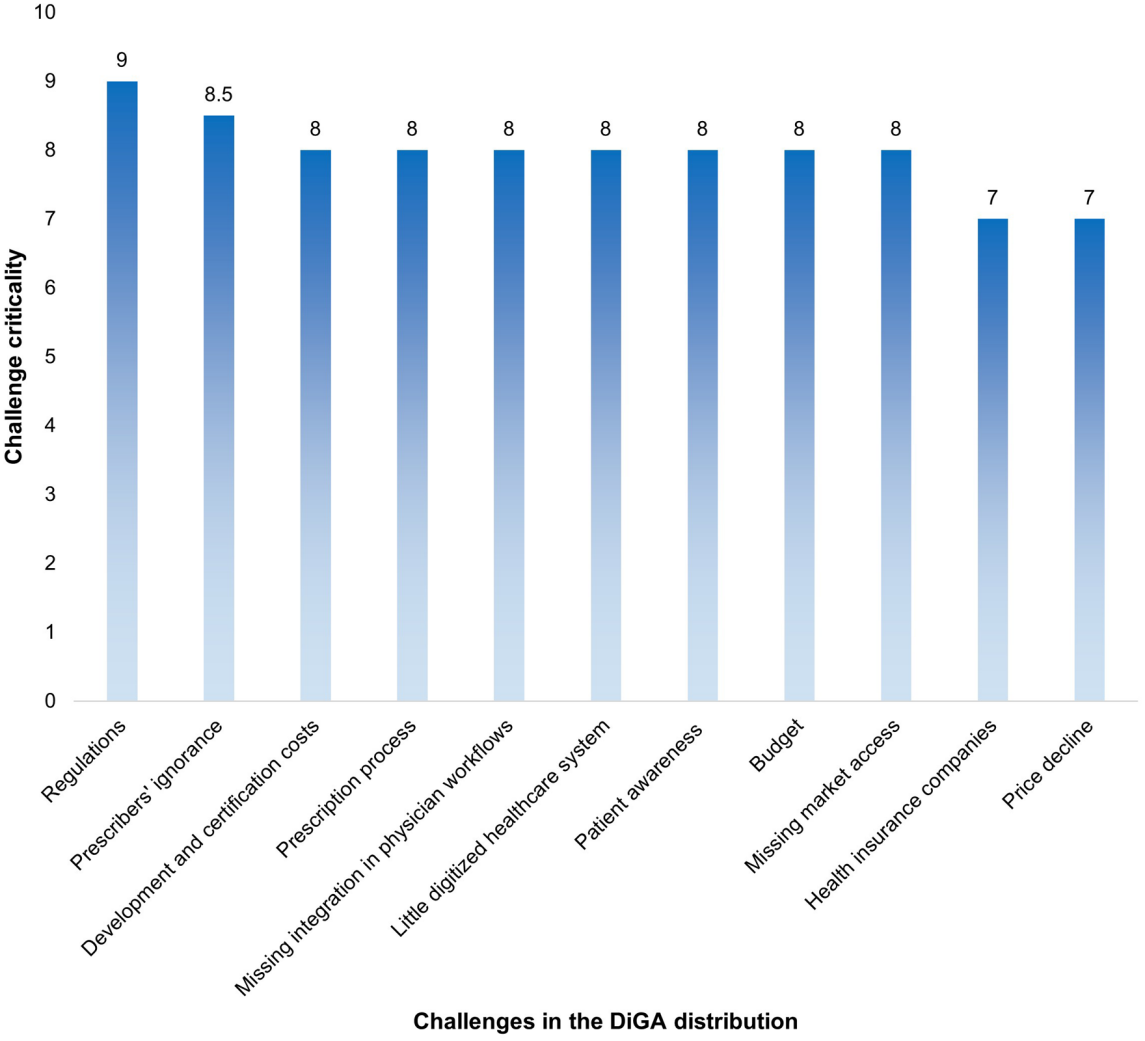
Challenges in DiGA distribution – overview.

Several additional challenges, each receiving a median rating of 8, were highlighted as significant barriers to DiGA success, as shown in the Supplemental S3 and Figure 6:

#### Future Scenarios for the Development of the DiGA Market

The survey results indicated several equally probable scenarios for the future of the DiGA market, each receiving a median rating of 8 out of 10:

- Market consolidation (a process already underway)
- Stricter regulatory requirements in data protection, information security, interoperability, and evidence
- Integration of AI in DiGA
- Improvement in the financial situation of DiGA startups
- DiGA is becoming part of standard care and increasing inclusion in Disease Management Programs (DMPs)
- Declining prices for DiGA

A comprehensive list of additional future scenarios is provided in the Supplemental Materials S3.

### Expectations of DiGA Integration into Standard Medical Care

43% of the 84 survey participants expect DiGA to become an integral part of standard medical care in the next 5 to 10 years. A proportion of survey participants (27%) believed that this transition could occur even sooner, within the next 3 to 5 years. However, a minority of 12% (*n* = 10) expressed skepticism, stating that DiGA would never become a standard component of medical care. A detailed breakdown of all responses is provided in the Supplemental Materials.

### Comparative Analysis of High Prescription Volume, Medium Prescription Volume and Low Prescription Volume DiGA Manufacturers

In addition to identifying key success factors, this study tested the hypothesis that DiGA manufacturers with high prescription volumes (>15 000) differ from those with moderate (2000-15 000) and low (<2000) prescription volumes in their evaluation of critical success factors. The categorization into low, moderate, and high prescription volumes was based on regulatory cut-off values and established market distributions rather than statistical clustering. Especially the threshold of 2000 prescriptions was selected due to its direct relevance for pricing and reimbursement decisions in the DiGA system.^28^

**Figure 7.**
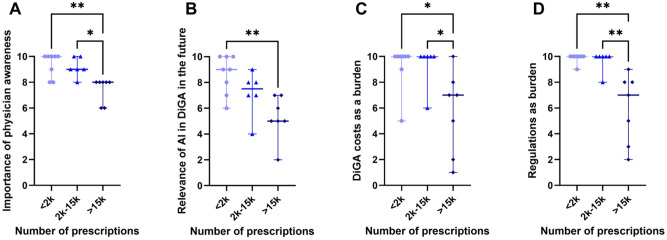
Differences in questionnaire scoring: (A) Difference in scoring the factor “importance of physician awareness” between low, medium and high prescription volumes (median_<2k_ = 10 vs median_>15k_ = 8, *p* < .01; median_2k-15k_ = 8, *p* < .01; median_2k-15k_ = 9 vs median_>15k_ = 8, *p* < .05), (B) differences in scoring the future scenario “relevance of AI DiGA future” between high, medium and low prescription volumes (median_2k_ = 9 vs median_>15k_ = 5, *p* < .01), (C) differences in scoring the challenge “DiGA development and certification cost” between high, medium low prescription volumes (median_<2k_ = 10 vs median_>15k_ = 7, *p* < .05; median_2k-15k_ = 10 vs median_>15k_ = 7, *p* < .05), (D) differences in scoring the challenge “regulatory” between high, medium and low prescription volumes (median_<2k_ = 10 vs median_>15k_ = 7, *p* < .01; median_2k-15k_ = 10 vs median_>15k_ = 7, *p* < .01). Significance levels: * < 0.05, ** < 0.01.

Across multiple categories, DiGA manufacturers with lower prescription volumes consistently rated factors as more important compared to those with higher prescription volumes. Manufacturers with fewer prescriptions rated the factors in Figure 7 significantly higher:

1. Physician awareness 
  The importance of strong presence and training at the point of care (doctors, therapists) to increase awareness and promote solution adoption was emphasized more by low-prescription manufacturers.
2. Relevance of AI in DiGA
   While AI-based DiGA were acknowledged as increasingly relevant in the interviews, lower-prescription volume manufacturers placed greater emphasis on regulatory challenges potentially hindering their adoption.
3. Development and certification costs as a burden 
  The financial burden of certification and prescription-related expenses was rated as a greater challenge by lower-prescription manufacturers, particularly regarding scalability in the B2C sector.
4. Regulations as a burden
   Smaller manufacturers perceived increasing regulatory demands for studies and declining DiGA prices as more obstructive to innovation and market entry.

## Discussion

The findings of this study align with existing literature on multiple key factors influencing DiGA success and adoption. Several elements identified in the survey correspond with prior research, reinforcing their relevance within the digital health ecosystem.

### Key Success Factors for DiGA Distribution

Key success factors were defined as the main factors that determine whether a business succeeds or fails based on the number of prescriptions.^29^ The findings highlight the multi-dimensional nature of DiGA success, reinforcing the necessity of strong clinical validation, strategic networking, and targeted awareness-building at the point of care as core components of an effective market entry strategy. Sources from the literature on software as a medical device and DTx substantiate the results on evidence,^30,31^ network management^32^ and the physician’s awareness.^33^ CME Training events were mentioned as a success factor, marketing strategy and key service offered by DiGA manufacturers. Trainings for physicians were further supported by survey findings, where 88% of physicians expressed interest in specialized digital health education, including CME-certified training.^34-36^

### Key Marketing and Sales Strategies for DiGA Distribution

We defined key marketing and sales strategies as DiGA manufacturer’s integrated approach to marketing activities and resources aimed at achieving high prescription volumes.^37^ The results emphasize the relevance of the right access to physicians, targeted prescriber targeting, and improvement of conversion rates. These findings are consistent with the top success factors. In particular, the combination of the factor of “awareness at the point of care” and the “right access to prescribers” as well as the “improvement of conversion rates“ through prescription services, is supported by current market observations. One prominent implementation of the mentioned combination is the use of fax-based communication targeting physicians, as applied by the DiGA manufacturer Oviva. Despite ethical concerns and outdated communication channels, its effectiveness is confirmed by recent prescription data. According to the official DiGA report published by the GKV on April 1, 2025, Oviva accounts for 17% of all DiGA prescriptions, totaling over 146 600 prescriptions, thereby clearly outperforming the second most prescribed DiGA, Vivira, with 91 200 prescriptions.^3^ This market dominance validates that direct physician engagement through familiar communication formats can succeed, even via outdated channels.

### Key Components and Features of Successful DiGA

The key components and features were defined as the characteristics and associated services offered by manufacturers. The results indicate: the success of a DiGA depends on a combination of different features, characteristics and services. First, a user-centered product that visibly benefits both patients and physicians.^31^ Second, seamless integration into existing care structures^31^ and prescription support systems. Although this is not a top priority, the benefits for physicians remain relevant, as 39% believe that digitization will reduce their workload.^31,34^ Improving DiGA adherence and patient access and success remains a critical factor for successful commercialization. Elements and services at the macro level, such as embedding DiGA in existing care frameworks, simplified prescribing and activation workflows and pay-for-performance models, have been proposed as potential solutions in the interviews and current research.^36^

### Key Challenges in DiGA Market Success

Overall, the findings underscore the regulatory, economic, and structural barriers that must be addressed to ensure the sustainable growth and adoption of digital health applications. Regulatory hurdles were identified as the most significant barrier to DiGA adoption, reflecting previous concerns regarding compliance with TR 03161, DVG, DigiG, DiGA-V, the DiGA guideline, BfArM data protection policies, the AI Act, and MDR. These regulatory frameworks create uncertainty and introduce significant challenges for manufacturers.^35^

The lack of physician awareness about DiGA was identified as the second most pressing barrier in this study, mirroring the findings of Cirkel et al, who also highlighted this issue as a major obstacle to DiGA adoption.^34,35^ The results of Dahlhausen et al showed that physicians saw substantial barriers associated with prescribing DiGA, most importantly, a lack of information, uncertainties regarding therapeutic benefits and medical evidence, and technical concerns.^35^

### Future Scenarios for the Development of the DiGA Market

The assessment of future scenarios is very balanced. The development of regulation will continue to grow in line with the ever-increasing requirements of the past, but it will also become increasingly complex in combination with new technological developments. The AI Act is a prime example of this. For AI-based DiGA, compliance becomes even more complex, particularly for stochastic machine learning models, which evolve dynamically.^38,39^ The Medical Device Regulation (MDR) mandates that software products demonstrate appropriate validation, but self-learning software can alter performance over time. If these changes exceed a defined threshold, they may necessitate a new conformity assessment, posing regulatory and commercialization challenges.^38^

Other scenarios include the aforementioned consolidation of the market and the ongoing tense financial situation for DiGA start-ups.^40^ Reports from the German Digital Healthcare Association (Spitzenverband Digitale Gesundheitsversorgung, SVDGV) describe an optimistic view of the willingness of investors and VCs to invest in DiGA.^41^

### Discussion on the Comparative Analysis

The findings of the comparative Analysis of high prescription volume, medium prescription volume and low prescription volume DiGA Manufacturers suggest that DiGA manufacturers with fewer prescription volumes tend to perceive more challenges and rate success factors and strategies higher in importance than those with a strong market presence. This could indicate that manufacturers with lower prescription volumes experience greater market entry barriers like new regulations and rely more heavily on these factors to improve their positioning.

This analysis further reveals a notable difference in the distribution of factor scoring between high prescription volume and low prescription volume DiGA manufacturers. While manufacturers with lower prescription volumes exhibit relatively uniform scoring across factors (*SD* = 2.2), those with higher prescription volumes demonstrate a broader variance in their responses (*SD* = 2.7). One survey participant attributed this divergence to the strong dependence of factor relevance on the specific indication, associated market dynamics, and medical environment. This observation is reinforced by multiple statements from the expert interviews, suggesting that the relative importance of success factors may vary considerably across specific indications. A thorough investigation into the prescription volumes on whole indication categories did not yield any significant findings. This is analogous to the present GKV DiGA report, which states that there will be prescription-relevant DiGA manufacturers in each indication category.^3^ Nevertheless, a discernible distinction emerges in the category of hormones and metabolism, as evidenced by the prescription volumes of Oviva and Zanadio, both of which are indicated for the treatment of obesity.^3^ This hypothesis warrants further investigation in future research to assess the extent to which indication-specific market conditions influence DiGA success strategies and adoption barriers.

### Study Limitations

One limitation of this study is the sample size of the expert interviews. However, when compared to previous research in a similar setting, the sample size of *n* = 32 exceeds that of a prior study (*n* = 19).^36^ This increase is largely due to the growing number of DiGA manufacturers entering the market since the earlier study. While this provides a broader perspective, future studies should aim to further expand the sample size to ensure even greater generalizability. A methodological limitation of this study is the absence of an established and validated interview instrument for this research context. Although the guide was carefully self-developed and refined through pilot testing to enhance its rigor and clarity, limitations regarding generalizability and reproducibility may nevertheless remain. Additionally, construct validity cannot be achieved with the questionnaire as there are neither convergent nor discriminant tests that measure the DiGA construct. Only 1 interviewer conducted the interviews. This could have led to subjective bias. A further limitation is the partial overlap between interview and survey participants, which may introduce a degree of response dependency. The validity of the content of the questionnaires is given based on technical and logical considerations, which were also checked by means of the interviews. The evaluation objectivity of the questionnaires is ensured by coding using a coding plan. Although the survey sample size was sufficient for exploratory analysis within the specialized DiGA ecosystem, the relatively small number of respondents limits the generalizability of the quantitative findings. The survey participants have previously noted that the survey itself requires a substantial degree of DiGA expertise, as it asks for detailed legal, technical, and industry-specific assessments in some cases. They have further indicated that individual factors and strategies are influenced by the indication for which the DiGA is intended. Consequently, normative studies for DiGA in the same indication are deemed to be highly recommended for future research endeavors.

## Conclusion

The present study provides a comprehensive analysis of the critical success factors, marketing and sales strategies, key product components and processes, and major challenges influencing the adoption and commercialization of DiGA.

1. Clinical evidence, physician awareness, and access to the right prescribers as primary success factors and strategies 
  The results indicate that robust clinical evidence, strong professional networks, and physician awareness at the point of care are the most critical determinants of DiGA success. High levels of clinical validation with recognized endpoints enhance trust and credibility in the medical field. Additionally, targeted engagement with healthcare professionals fosters broader DiGA adoption and prescription rates. With regard to the product, a DiGA must be user-oriented and clearly demonstrate success for patients and prescribers.
2. Regulatory burdens and market challenges as key barriers 
  The study highlights increasing regulatory requirements and high development and certification costs that come with it, physician unawareness and the DiGA prescription process as the most pressing barriers to market success. Compliance with MDR, AI Act, and DiGA guidelines is particularly difficult for AI-based or blended care applications. Additionally, the relationship with public health insurers was identified as a structural issue, with some manufacturers reporting active resistance, the deliberate spreading of misinformation and restrictive reimbursement practices.
3. Differentiation in perceived importance of success factors based on prescription volume
   A comparative analysis between high-prescription and low-prescription DiGA manufacturers revealed significant differences in the scoring of key factors. Manufacturers with lower prescription volumes tend to assign consistently high importance to multiple success factors, whereas high prescription volume manufacturers show greater variability in their assessments. This suggests that the relevance of success factors is highly indication-dependent, with market conditions and medical environments shaping individual commercialization strategies.

### Scientific and Practical Implications

For academia, this study contributes to the growing body of research on digital therapeutics and digital health commercialization by systematically identifying and quantifying the factors that drive DiGA success. The findings provide a foundation for future research, particularly regarding indication-specific commercialization strategies, cost-effectiveness analyses, and regulatory impact assessments.

For DiGA manufacturers, these insights offer practical guidance for optimizing market entry and scaling strategies. The importance of physician engagement, strategic regulatory navigation, and differentiated go-to-market approaches is underscored as essential for long-term success.

### Future Research Directions

While this study provides a structured framework for understanding DiGA success, future research should explore longitudinal analyses of successful DiGA companies, investigate the economic impact of regulatory requirements, and further examine the differentiation of commercialization strategies based on therapeutic area and indication. Additionally, further validation in an international context could help assess whether the identified factors are specific to the German DiGA system or applicable to broader digital health ecosystems.

## Supplemental Material

sj-docx-1-inq-10.1177_00469580261433432 – Supplemental material for Accessing the Digital Health Application (DiGA) Market: Key Success Factors, Market Barriers and Strategies for Sustainable Adoption Supplemental material, sj-docx-1-inq-10.1177_00469580261433432 for Accessing the Digital Health Application (DiGA) Market: Key Success Factors, Market Barriers and Strategies for Sustainable Adoption by Lukas Schramm, Nadine Cebulla, Christian Greis and Claus-Christian Carbon in INQUIRY: The Journal of Health Care Organization, Provision, and Financing

sj-docx-2-inq-10.1177_00469580261433432 – Supplemental material for Accessing the Digital Health Application (DiGA) Market: Key Success Factors, Market Barriers and Strategies for Sustainable Adoption Supplemental material, sj-docx-2-inq-10.1177_00469580261433432 for Accessing the Digital Health Application (DiGA) Market: Key Success Factors, Market Barriers and Strategies for Sustainable Adoption by Lukas Schramm, Nadine Cebulla, Christian Greis and Claus-Christian Carbon in INQUIRY: The Journal of Health Care Organization, Provision, and Financing
