# Supplementary material for: Accessing the Digital Health Application (DiGA) Market: Key Success Factors, Market Barriers and Strategies for Sustainable Adoption
Source: Inquiry. 2026 Apr 10;63:00469580261433432. doi: 10.1177/00469580261433432 (PMC13070170; doi:10.1177/00469580261433432)
Supplement: sj-docx-1-inq-10.1177_00469580261433432 – Supplemental material for Accessing the Digital Health Application (DiGA) Market: Key Success Factors, Market Barriers and Strategies for Sustainable Adoption [file sj-docx-1-inq-10.1177_00469580261433432.docx]

# S1 Interview Questionnaire

1. Working enviroment:

German:Beschreiben Sie kurz Ihr Arbeitsumfeld und was Ihre Tätigkeit ausmacht?

English: Briefly describe your working environment and what your job is about?

_____________________________________________________________________________

_____________________________________________________________________________

2. Introduction and Background:

German: Können Sie uns etwas über Ihre Erfahrung in der Vermarktung digitaler Gesundheitsanwendungen erzählen?

English: Could you provide insights into your experience in the distribution of digital health applications?

_____________________________________________________________________________

_____________________________________________________________________________

3. DiGA Success Factors:

German: Welche fünf Faktoren tragen Ihrer Meinung nach am meisten zum Erfolg bei der Vermarktung digitaler Gesundheitsanwendungen bei?

English: In your opinion, what five factors have contributed the most to the success of the distribution of digital health applications?

_____________________________________________________________________________

_____________________________________________________________________________

4. DiGA Distribution Strategies:

German: Welche top drei Strategien haben sich als besonders wirksam bei der Vermarktung digitaler Gesundheitsanwendungen erwiesen?

English: What top three strategies have proven most effective in marketing digital health applications?

_____________________________________________________________________________

_____________________________________________________________________________

5. Key Components and Features of successfull DiGA (Manufacturers):

German: Gibt es bestimmte Schlüsselkomponenten oder Merkmale, die Ihrer Meinung nach entscheidend für den Erfolg von digitalen Gesundheitsanwendungen sind?

English: Are there specific key components or features that you believe are crucial for the success of digital health applications?

_____________________________________________________________________________

_____________________________________________________________________________

6. Key Challenges and Barriers for DiGA Distribution:

German: Was sind die fünf größten Herausforderungen und Probleme bei der Vermarktung digitaler Gesundheitsanwendungen und wie gehen Sie damit um?

English: What are the five biggest challenges and problems in marketing digital health applications and how do you deal with them?

_____________________________________________________________________________

_____________________________________________________________________________

7. Future Scenarios:

German: Bei welchen dieser Herausforderungen oder Problemen erwarten sie, dass diese in den nächsten 10 Jahren gelöst sein werden und welche neuen Probleme kommen vielleicht dazu?

English: Which of these challenges or problems do you expect to be solved in the next 10 years, and which new problems might arise?

_____________________________________________________________________________

_____________________________________________________________________________

8. Outlook:

German: Wann denken Sie, dass Digitale Gesundheitsanwendungen (DiGA) in der Gesundheitsversorgung als Standard etabliert sein werden? Oder wo sind DiGA schon Standard?

English: When do you envision Digital Health Applications (DiGA) becoming established as a standard in healthcare provision? Or where are DiGA already standard?

_____________________________________________________________________________

_____________________________________________________________________________
