# Supplementary material for: Accessing the Digital Health Application (DiGA) Market: Key Success Factors, Market Barriers and Strategies for Sustainable Adoption
Source: Inquiry. 2026 Apr 10;63:00469580261433432. doi: 10.1177/00469580261433432 (PMC13070170; doi:10.1177/00469580261433432)
Supplement: sj-docx-2-inq-10.1177_00469580261433432 – Supplemental material for Accessing the Digital Health Application (DiGA) Market: Key Success Factors, Market Barriers and Strategies for Sustainable Adoption [file sj-docx-2-inq-10.1177_00469580261433432.docx]

# S2 Further information on interviews and survey

## Questionnaire development

Based on feedback from two experts during pilot testing, the interview guide was revised to primarily include closed ended questions. Although open-ended questions can generate new ideas^1^, they were seen as less effective in providing a comprehensive overview of the topic and could lead the discussion away from key points. The use of closed-ended questions limited the range of potential responses, narrowing the scope of results. However, this approach ensured that participants provided more focused and specific information, aligning with the study’s primary objectives.

## Conducting the interviews

For the interview, face-to-face approaches were conducted personally, utilizing Computer Assisted Personal Interviews (CAPI). To ensure the highest possible level of objectivity in the interview process, all interviews were conducted using a standardized approach. The same equipment was consistently used, and the interviewer's attire and background were kept uniform across all sessions. To maintain consistency in the interpretation of the data, all semi-structured interviews were conducted by a single interviewer.

Including experts beyond DiGA manufacturers in the interview process was methodologically justified to capture diverse perspectives on success factors and challenges, and to ensure a sufficient sample size, given the relatively small number of active DiGA manufacturers in the market. This resulted in data saturation.

The ancillary criterion of the economy was ensured through the straightforward administration and swift analysis facilitated by dedicated tools, specifically Microsoft Teams, for conducting interviews and transcription^2^.

## Survey development

To optimize the survey's professionalism and coherence, strategically positioned filter questions and a progress bar serve to heighten transparency, potentially mitigating attrition rates^3,4^. Furthermore, the attrition rate was intended to be mitigated through a concise completion time of 20-25 minutes and a limited set of 14 questions strategically designed to ensure brevity^3^.

We distributed the test among two cohorts: one composed of individuals possessing proficiency in questionnaire construction and a second group representing participants capable of articulating issues pertaining to comprehensibility, usability, and duration of completion from a respondent's standpoint. Pertinent observations gleaned from the pretest participants were discussed and adopted to each survey page. The pretest phase thus assumed a pivotal role in empirical quality assurance^5^.

Additional facets of quality assurance encompass the supplementation of open-ended questions alongside closed-choice queries, thereby enabling a more in-depth exploration and validation of the selected options. Furthermore, a variation in Likert scale presentation, involving an alternating arrangement of poles from *strongly agree* to *strongly disagree*, was implemented to mitigate response biases.

The questions in the survey were structured in the same way as in the interviews to avoid distorting the results and ensure consistency in the answers. The individual factors to be assessed within the questions were organized according to the frequency with which they were mentioned in the expert interviews. The most frequently mentioned were in first place, and those with the fewest mentions were in last place. The responses were evaluated using a Likert scale. Due to the large number of mentions, only factors that were mentioned by at least three experts were listed.

In tandem with the pretest, a technical examination of the questionnaire was conducted as part of the comprehensive quality control process^6^. The technical examination encompasses a thorough assessment of data export functionality, compatibility across various browsers, responsiveness, display optimization for mobile devices and systems (iOS & Android), as well as the secure storage of responses and their respective formats. The survey blocks followed the same structure as the interviews, use automatic filters, require mandatory responses, and collect processing metadata^3,6^. An additional merit inherent in online surveys is the liberation from the necessity of the physical presence of an experimenter during administration, thereby affording heightened flexibility in the data collection process^7^.

## Characteristics of the data collection via survey

The value of the interviews and the survey stems from a practical need and the current lack of standardized tests for evaluating success factors in this area. This fulfills an additional criterion of concordant validity. Comparability was ensured through the inclusion of similar questions in both the interview and the survey. In addition to nominal scales for categorization, interval scales are predominantly employed to gather rank data, facilitating the prioritization of success factors and enabling statistical means and variance analyses.

Leveraging the presumed digital acumen inherent in their professional roles, online tools are deemed more efficacious for engaging this target group compared to conventional paper-and-pencil surveys. Exclusion of patient involvement was grounded in the acknowledgment of distinct interests in DiGA utilization and prescription within this demographic. We increased the participation rate further through incentives in the form of information about the results^3,6^.

# S3 Further information on Results

## Key success factors for DiGA distribution

Table 1: (1) Key Success Factors for DiGA distribution

| Mentioned in expert interview | Scoring (median) in questionnaire | Scoring (mean) in questionnaire | Factor | Description |
| --- | --- | --- | --- | --- |
| 13 | 9 | 8.5 | Clinical evidence | Repeated confirmation of clinical evidence with recognized endpoints enhances credibility and acceptance in the medical field |
| 14 | 9 | 8.5 | Existing professional network | Building trusted relationships with specialists strengthens long-term collaboration and facilitates access to new markets |
| 9 | 9 | 8.3 | Awareness at the point of care | Strong presence and training at the point of care (prescribers, therapists) increase awareness and promote solution adoption |
| 3 | 8 | 8 | Familiar format for marketing material | Presenting information in a format familiar to prescribers increases acceptance and trust in the solution |
| 4 | 8 | 7.8 | Selected indication | Targeted selection of the appropriate patient group and indication increases the effectiveness and market acceptance of the solution |
| 11 | 8 | 7.7 | Unmet medical need | Solving an unmet medical need is critical for market success and sustained demand |
| 5 | 8 | 7.6 | Great product | Ease of use and high product customizability, through an appealing UI and gamification, boost user satisfaction and retention |
| 6 | 8 | 7.4 | Patient suffering | Patient suffering in specific indications can significantly boost demand and acceptance of the solution |
| 3 | 8 | 7.4 | Omnichannel approach | A well-defined sales strategy and the use of omnichannel approaches improve market penetration and customer retention |
| 6 | 8 | 7.3 | Maintain KOL network | Continuous engagement with key opinion leaders (KOL) in the relevant field enhances credibility and promotes solution adoption |
| 3 | 8 | 7.3 | USP | Clearly defining the unique selling proposition (USP) of the DiGA is crucial to stand out from competitors |
| 3 | 8 | 7 | Presence at trade fairs | Regular attendance at trade shows, conferences, and congresses promotes engagement with the professional community and increases market visibility |
| 4 | 7 | 7 | Patient activability | Patient activation, depending on the indication, is crucial for the successful implementation and use of the solution |
| 5 | 7 | 7 | Optimized B2C business | Optimizing patient outreach through alternative channels and platforms can increase the reach and effectiveness of marketing efforts |
| 4 | 7 | 6.5 | First to market | Being first to market provides a clear competitive advantage and allows for early market share acquisition |
| 6 | 7 | 6.4 | Pricing strategy | A sound pricing strategy and flexible pricing model are essential to ensure market access and profitability |
| 9 | 6.5 | 6.5 | Large patient collective | Focusing on indications with a large patient population maximizes potential market share and prescription rates |
| 4 | 6.5 | 6 | CME training | Regular CME events and training for prescribers promote expertise and willingness to adopt the DiGA |
| 6 | 6 | 6.3 | In-house sales team | Building an in-house sales force strengthens brand control and enables targeted customer engagement |
| 5 | 6 | 6 | Early launched Marketing activities | Starting marketing efforts before approval allows for faster market entry and increases awareness of the DiGA during the development phase |
| 3 | 6 | 6 | Productportfolio | Combining the DiGA with an existing product portfolio facilitates market entry and strengthens the sales strategy |
| 9 | 6 | 5.8 | Early started pharma-coop | Early sales partnerships with pharma and medtech partners ensure rapid market penetration and expand the distribution network |

Figure 1: (1) Key Success Factors for DiGA distribution

## Key marketing & sales strategies for DiGA distribution

Table 2: (2)Key marketing & sales strategies for DiGA distribution

| Mentioned in expert interview | Scoring (median) in questionnaire | Scoring (mean) in questionnaire | Strategy | Description |
| --- | --- | --- | --- | --- |
| 13 | 9 | 8 | Choice of right access to physicians | Choosing the right access to prescribers and providing evidence-based information through publications, conferences, and practice visits enhances trust and visibility |
| 5 | 8 | 7.7 | Prescriber targeting | Targeting the appropriate specialist group and focusing on prescriber-centric marketing maximizes prescription rates and market penetration |
| 8 | 8 | 7.4 | Improvement in Conversion Rates | Measures such as diagnostic aids, telemedicine, and prescription services make it easier for patients to redeem codes and improve data collection for targeted adjustments |
| 6 | 8 | 7.3 | Optimized patient journey | A marketing strategy aligned with the patient journey |
| 4 | 8 | 6.9 | Partner | Collaboration with relevant partners like care centers, associations, and other DiGA manufacturers increases reach and market presence in specific indications |
| 6 | 7 | 6.7 | Patient groups | Activating well-organized patient groups and using community approaches combined with social influencing maximize reach and engagement, especially for chronic conditions |
| 4 | 7 | 6.7 | Blended Care | Blended care approaches with frequent monitoring and compensated prescriber-patient interactions improve treatment outcomes and patient retention |
| 6 | 7 | 6.3 | CME training events | Online CME webinars and training increase reach and help engage digital practices and specialists more effectively |
| 18 | 7 | 6 | Sales cooperations | Sales partnerships with pharmaceutical companies that have existing networks with prescribers are crucial for start-ups to accelerate market entry |
| 4 | 6.5 | 6 | DiGA & Drug combination | Combining the DiGA with pharmaceutical products tailored to a specific group of prescribers |

Figure 2: (2) Key marketing and sales strategies for DiGA distribution

## Key Components and features of successful DiGA

Table 3: (3) Key Components and features of successful DiGA

| Mentioned in expert interview | Scoring (median) in questionnaire | Scoring (mean) in questionnaire | Componente, key Feature | Description |
| --- | --- | --- | --- | --- |
| 6 | 9 | 8.4 | Patient success | Demonstrating patient success, value, and effectiveness to prescribers and patients encourages acceptance and long-term use of the DiGA |
| 23 | 9 | 8.3 | Great Product | User-centered design with stable functionality, ease of use, motivational elements that promote adherence, and personalized features supported by gamification and a well-structured user journey |
| 5 | 8 | 8.1 | Physician benefits | Offering benefits for prescribers, such as time savings, addressing pain points, and compensation, increases their willingness to prescribe the DiGA |
| 4 | 8 | 8.1 | Embedded in existing care concepts | Integrating the DiGA into existing care frameworks and addressing unmet medical needs promote acceptance |
| 9 | 8 | 8 | Prescription service | Efficient prescription services and well-organized customer support simplify the use of the DiGA and enhance customer satisfaction |
| 13 | 8 | 7.9 | Evidence | Additional validation through real-world data (RWD), patient-reported outcome measures (PROM), and high-quality randomized controlled trials (RCT) boosts credibility |
| 4 | 8 | 7.6 | Marketing Budget | Marketing Budget |
| 3 | 8 | 7.5 | Customized language | Using simple, understandable, and audience-appropriate language facilitates communication and acceptance among prescribers and patients |
| 3 | 8 | 7.4 | Prescriber-centered marketing | A sales focus on prescribers enhances prescription willingness and improves market penetration |
| 6 | 7 | 6.6 | Large patient collective | Selecting a mass-market indication with a large patient population maximizes market opportunities and growth potential |
| 3 | 7 | 6.3 | CME training | Offering continuing education and CME training for prescribers enhances expertise and promotes the prescription of the DiGA |
| 3 | 6 | 6 | DiGA Price | Differentiating through an attractive cost-benefit ratio and lower price increases the DiGA’s competitiveness |

Figure 3: (3) Key Components and features of successful DiGA

## Key Challenges in DiGA Market Success

Table 4: (4) Key Challenges in DiGA Market Success

| Mentioned in expert interview | Scoring (median) in questionnaire | Scoring (mean) in questionnaire | Challenge or barrier | Description |
| --- | --- | --- | --- | --- |
| 15 | 9 | 8.1 | Regulations | Growing regulatory requirements for studies, data protection, information security and interoperability |
| 24 | 8.5 | 8 | Prescribers' ignorance | Many prescribers are unaware of or resistant to DiGA solutions, leading to slow market penetration with only early adopters reached, while the majority remains untapped |
| 4 | 8 | 7.7 | Development and certification costs | High development costs for certifications and prescription expenses are particularly unscalable in the B2C sector, threatening profitability |
| 8 | 8 | 7.6 | Prescription process | Low conversion rates in DiGA prescription redemption and cumbersome prescription submission processes hinder adoption |
| 3 | 8 | 7.6 | Missing integration in physician workflows | Lack of DiGA integration into prescribers' workflows and absence of feedback loops reduce effectiveness and adherence |
| 3 | 8 | 7.5 | Little digitized healthcare system | Insufficient digitalization in healthcare and the telematics infrastructure hinders the integration of DiGA solutions and cloud providers |
| 9 | 8 | 7.3 | Patient awareness | The DiGA is not yet well-known among patients, limiting its usage and demand |
| 15 | 8 | 7 | Budget | Start-ups often struggle to secure sufficient capital for expensive marketing and scaling efforts, slowing their growth |
| 5 | 8 | 6.9 | Missing market access | Many DiGA companies lack direct access to prescribers or relevant networks, making prescription and acceptance difficult |
| 20 | 7 | 6.8 | Health insurance companies | Public health insurers (GKV) actively oppose DiGA products, making market access difficult and hampering reimbursement options |
| 4 | 7 | 6.6 | Price decline | Falling prices in the DiGA market make it difficult for manufacturers to develop and maintain sustainable business models |

Figure 4: (4) Key Challenges in DiGA Market Success

## Future Scenarios for the Development of the DiGA Market

Table 5: (5) Future Scenarios for the Development of the DiGA Market

| Mentioned in expert interview | Scoring (median) in questionnaire | Scoring (mean) in questionnaire | Scenario | Description |
| --- | --- | --- | --- | --- |
| 4 | 8 | 8 | Financial situation | Budget constraints will remain a persistent challenge for DiGA start-ups and manufacturers |
| 4 | 8 | 7.8 | Market consolidation | Market consolidation will occur as less effective DiGA are removed, increasing pressure on providers to offer high-quality solutions |
| 15 | 8 | 7.6 | Strict regulations | Increasing regulatory demands for data protection, interoperability, and evidence will raise barriers to market entry |
| 6 | 8 | 7.25 | AI in DiGA | AI-based DiGA will become more common, but regulatory challenges may hinder its rapid implementation |
| 3 | 8 | 7.1 | Part of standard care (DMP) | The integration of DiGA into guidelines and treatment programs will boost acceptance but require constant quality and evidence |
| 8 | 8 | 6.9 | Falling prices | Continued price drops will exert significant pressure on DiGA manufacturers to optimize their cost structures |
| 8 | 7 | 7.2 | Competition | With increasing competition, DiGA companies must find innovative ways to differentiate themselves |
| 16 | 7 | 7 | DiGA awareness | Market penetration and awareness among prescribers and patients increases |
| 5 | 7 | 7 | Demographic change | The generational shift will lead to greater DiGA acceptance and new demands for modern technologies |
| 9 | 7 | 6.8 | Improved interoperability | Interoperability - the building blocks for digitizing the healthcare system e-prescription, ePa, TI and DiGA come together |
| 7 | 7 | 6.6 | Internationalization | International standardization will facilitate access to larger markets but also increase global competition |
| Market Observation | 7 | 6.6 | More DiGA in MDR Iia | The majority of DiGAs in risk class I will move up to risk class IIa due to new regulatory interpretations |
| 8 | 7 | 6.4 | ePrescription | The improved submission process via e-prescription will increase conversion rates, but also raise expectations for DiGA provider efficiency |
| 3 | 7 | 6.25 | Deterioration in health insurance relationships | Worsening relations with health insurers could make access and reimbursement for DiGA more difficult |
| 9 | 5 | 5.1 | Improvement of health insurance relationships | Collaboration with health insurers may improve, leading to better reimbursement terms and greater acceptance |

Figure 5: (5) Future Scenarios for the Development of the DiGA Market

# References

1. Möhring W, Schlütz D. *Handbuch standardisierte Erhebungsverfahren in der Kommunikationswissenschaft*. Wiesbaden: Springer VS, 2013.

2. Bühner M. *Einführung in die Test- und Fragebogenkonstruktion*. 3., aktualisierte und erw. Aufl. München: Pearson Studium, 2011.

3. Von Baur N, Florian MJ. Stichprobenprobleme bei Online-Umfragen. In: Jackob N, Schoen H, Zerback T (eds) *Sozialforschung im Internet*. Wiesbaden: VS Verlag für Sozialwissenschaften, pp. 109–128.

4. Von Pötschke M. Potentiale von Online-Befragungen: Erfahrungen aus der Hochschulforschung. In: Jackob N, Schoen H, Zerback T (eds) *Sozialforschung im Internet*. Wiesbaden: VS Verlag für Sozialwissenschaften, pp. 75–89.

5. Schnell R. *Survey-Interviews: Methoden standardisierter Befragungen*. Wiesbaden: Springer Fachmedien Wiesbaden. Epub ahead of print 2019. DOI: 10.1007/978-3-531-19901-6.

6. Kuckartz U, Ebert T, Rädiker S, et al. *Evaluation online: internetgestützte Befragung in der Praxis*. Wiesbaden: VS Verlag für Sozialwissenschaften, 2009.

7. Von Roessing T. Internet für Online-Forscher: Protokolle, Dienste und Kommunikationsmodi. In: Jackob N, Schoen H, Zerback T (eds) *Sozialforschung im Internet*. Wiesbaden: VS Verlag für Sozialwissenschaften, pp. 49–58.
